# Common myths and misconceptions about breast cancer causation among Palestinian women: a national cross-sectional study

**DOI:** 10.1186/s12889-023-17074-9

**Published:** 2023-11-29

**Authors:** Mohamedraed Elshami, Islam Osama Ismail, Mohammed Alser, Ibrahim Al-Slaibi, Roba Jamal Ghithan, Faten Darwish Usrof, Malak Ayman Mousa Qawasmi, Heba Mahmoud Okshiya, Nouran Ramzi Shaban Shurrab, Ibtisam Ismail Mahfouz, Aseel AbdulQader Fannon, Mona Radi Mohammad Hawa, Narmeen Giacaman, Manar Ahmaro, Rula Khader Zaatreh, Wafa Aqel AbuKhalil, Noor Khairi Melhim, Ruba Jamal Madbouh, Hala Jamal Abu Hziema, Raghad Abed-Allateef Lahlooh, Sara Nawaf Ubaiat, Nour Ali Jaffal, Reem Khaled Alawna, Salsabeel Naeem Abed, Bessan Nimer Ali Abuzahra, Aya Jawad Abu Kwaik, Mays Hafez Dodin, Raghad Othman Taha, Dina Mohammed Alashqar, Roaa Abd-al-Fattah Mobarak, Tasneem Smerat, Shurouq I. Albarqi, Nasser Abu-El-Noor, Bettina Bottcher

**Affiliations:** 1 Division of Surgical Oncology, Department of Surgery, University Hospitals Cleveland Medical Center, 11100 Euclid Avenue, Cleveland, OH 44106 USA; 2 Ministry of Health, Gaza, Palestine; 3 United Nations Relief and Works Agency for Palestine Refugees (UNRWA), Gaza, Palestine; 4 Almakassed Hospital, Jerusalem, Palestine; 5 Faculty of Medicine, Al-Quds University, Jerusalem, Palestine; 6 Department of a Medical Laboratory Sciences, Faculty of Health Sciences, Islamic University of Gaza, Gaza City, Palestine; 7 Department of Medical Laboratory Sciences, Hebron University, Hebron, Palestine; 8 Al-Shifa Hospital, Gaza, Palestine; 9 Faculty of Medicine, Islamic University of Gaza, Gaza, Palestine; 10 Tulkarem Governmental Hospital, Tulkarem, Palestine; 11 Caritas Baby Hospital, Bethlehem, Palestine; 12 Department of Pharmacy, An-Najah National University, Nablus, Palestine; 13 Faculty of Dentistry, Al-Quds University, Jerusalem, Palestine; 14 Faculty of Medicine and Health Sciences, Palestine Polytechnic University, Hebron, Palestine; 15 Faculty of Pharmacy, Al-Azhar University of Gaza, Gaza, Palestine; 16 Faculty of Nursing, Islamic University of Gaza, Gaza, Palestine

**Keywords:** Breast cancer, Causation, Causes, Myths, Misconceptions, Health promotion, Health education, Palestine

## Abstract

**Background:**

The discussion about breast cancer (BC) causation continues to be surrounded by a number of myths and misbeliefs. If efforts are misdirected towards reducing risk from false mythical causes, individuals might be less likely to consider and adopt risk-reducing behaviors for evidence-based BC causes. This national study aimed to assess the awareness of BC causation myths and misbeliefs among Palestinian women, and examine the factors associated with having good awareness.

**Methods:**

This national cross-sectional study recruited adult women from government hospitals, primary healthcare centers, and public spaces in 11 governorates in Palestine. A modified version of the Cancer Awareness Measure-Mythical Causes Scale was used to collect data. The level of awareness of BC causation myths was determined based on the number of myths recognized to be incorrect: poor (0–5), fair (6–10), or good (11–15).

**Results:**

A total of 5,257 questionnaires were included. Only 269 participants (5.1%) demonstrated good awareness (i.e., recognizing more than 10 out of 15 BC mythical causes). There were no notable differences in displaying good awareness between the main areas of Palestine, the Gaza Strip and the West Bank and Jerusalem (5.1% vs. 5.1%). Having chronic disease as well as visiting hospitals and primary healthcare centers were associated with a decrease in the likelihood of displaying good awareness.

Myths related to food were less frequently recognized as incorrect than food-unrelated myths. ‘Eating burnt food’ was the most recognized food-related myth (*n* = 1414, 26.9%), while ‘eating food containing additives’ was the least recognized (*n* = 599, 11.4%). ‘Having a physical trauma’ was the most recognized food-unrelated myth (*n* = 2795, 53.2%), whereas the least recognized was ‘wearing tight bra’ (*n* = 1018, 19.4%).

**Conclusions:**

A very small proportion of Palestinian women could recognize 10 or more myths around BC causation. There is a substantial need to include clear information about BC causation in future educational interventions besides focusing on BC screening, signs and symptoms, and risk factors.

**Supplementary Information:**

The online version contains supplementary material available at 10.1186/s12889-023-17074-9.

## Background

Breast Cancer (BC) is the most common cancer and is the leading cause of cancer-related deaths among women worldwide. In 2020, The International Agency for Research on Cancer reported an estimated number of 2.26 million new BC cases, accounting for 24.5% of all female cancer cases, and about 684 thousand BC deaths, accounting for 15.5% of all female cancer-related deaths [1]. In Palestine, BC accounts for 35.6% of all reported female cancer cases, with estimated age-standardized incidence and mortality rates of 53.5 and 22.6 per 100,000 female population, respectively [2].

BC burden is heavier in low- and middle-income countries (LMICs) and its incidence is increasing due to higher trends for risk factors related to lifestyle modifications [3]. These factors include smoking, increased body mass index, physical inactivity, and changes in reproductive patterns (e.g., older age at first childbirth, fewer childbirths). Furthermore, BC mortality rates are higher in LMICs [3]. This could be attributed to diagnosis of BC at more advanced stages and difficulties in access to healthcare services [3, 4]. Primary prevention remains a cornerstone in mitigating BC burden [5]. Cancer awareness has been shown to be a major contributor to substantial improvement in cancer outcomes [6]. However, several studies demonstrated low awareness levels among Palestinian women about BC risk factors, symptoms, and screening methods [7–9].

Health beliefs are strong determinants of health behaviors [10], and accurate knowledge may play a key role in shaping protective health behaviors [11]. Around the world, the discussion about cancer causation, BC causation in particular, continues to be surrounded by a significant number of myths, such as stress, food additives, microwave ovens use, physical trauma, and exposure to electromagnetic frequencies [12–14]. If efforts are misdirected towards reducing risk from false mythical cancer causes, individuals might be less likely to consider and adopt risk-reducing behaviors for evidence-based BC causes [12, 14].

Therefore, this national cross-sectional study aimed to assess the level of awareness of BC causation myths and misbeliefs among Palestinian women and to compare it between women from the two main areas of Palestine, the West Bank and Jerusalem (WBJ) and the Gaza Strip. In addition, it aimed to analyze factors associated with having good awareness level of BC mythical causes.

## Methods

### Study design and population

This was a national cross-sectional study conducted between July 2019 and March 2020. The target population was adult (≥ 18 years) Palestinian women. Participants were excluded if they were visitors or patients admitted to the oncology departments, had a background in health sciences, were healthcare workers, had nationalities other than the Palestinian, or were unable to complete the questionnaire.

### Sampling size calculation

In 2019, the female population aging 15 years or older was 1,534,371 in Palestine [15]. With a confidence level of 95.0%, a type I error rate of 5.0% and an absolute error of 1.0%, the minimum sample size needed to detect a 10% good overall awareness of BC causation myths was 900 participants.

### Sampling methods

The Palestinian Ministry of Health has 466 primary healthcare centers; among them, 26 are of the highest level (i.e., level IV), which provides all primary care services. Out of the 26 primary healthcare centers, 17 are located in the WBJ and nine are located in the Gaza Strip. The Palestinian Ministry of Health also has 43 hospitals; 29 of them are in the West Bank and 14 are in the Gaza Strip. Of note, there are only 11 general hospitals with a bed capacity of more than 100: six in the West Bank and five in the Gaza Strip. There is no Ministry of Health hospital in Jerusalem. However, non-governmental organizations own three general hospitals with a bed capacity of more than 100 [16].

Convenience sampling was utilized to recruit women who fulfilled the eligibility criteria from 12 hospitals with bed capacity of more than 100, 11 level-four primary healthcare centers, and public spaces in 11 Palestinian governorates. These governorates included seven in the WBJ (Hebron, Nablus, Ramallah, Tulkarm, Bethlehem, Jenin, and Jerusalem) and four in the Gaza Strip (North of Gaza, Gaza, Middle Zone, and Khanyounis). Supplementary Table 1 summarizes the hospitals and primary healthcare centers included in this study. Public spaces included marketplaces, parks, malls, trade streets, mosques, transportation stations, and others. Recruiting participants from a variety of governorates and locations across Palestine was intended to increase the representativeness of the study cohort [8, 9, 17–27].

### Questionnaire and data collection

The Cancer Awareness Measure-Mythical Causes Scale (CAM-MYCS) [28] was adapted for the purpose of data collection. In a back-to-back translation, two bilingual healthcare experts, who were fluent in both English and Arabic, first translated the CAM-MYCS into Arabic and then two other bilingual healthcare experts back-translated it into English. All those healthcare experts had relevant expertise in BC, public health, and survey design. This was followed by assessing the questionnaire’s content validity by five independent healthcare professionals and researchers. A pilot study (*n* = 35) was then conducted to evaluate the clarity of the Arabic version of the questionnaire. The questionnaires of the pilot study were not included in the final analysis. Internal consistency was evaluated utilizing Cronbach’s Alpha, which reached an acceptable level of 0.74.

The questionnaire comprised two sections. The first section described the sociodemographic characteristics of participants, while the second section evaluated the prompt recognition of 15 myths around BC causation as being incorrect. Out of the 15 myths, 12 were adapted from the original CAM-MYCS. ‘Eating burnt food’, ‘using deodorant’ and ‘wearing tight bra’ were added as they were deemed important in the context of the Palestinian community. The original CAM-MYCS questions with correct/incorrect/unsure responses were changed into 5-point Likert scale questions (1 = "strongly disagree" to 5 = "strongly agree"). This was done to reduce the potential of participants answering questions at random. Responses with ‘disagree’ or ‘strongly disagree’ were considered correct; all others were considered incorrect. The awareness of myths about BC causation was evaluated using a scoring system that was utilized in previous studies [8, 9, 17–27]. The participant was given one point for each recognized myth to be incorrect. The total score was calculated and ranged from 0 to 15. The awareness level was determined based on the number of myths recognized to be incorrect: poor (0 to 5), fair (6 to 10), and good awareness (11 to 15).

Trained data collectors invited eligible participants to complete the questionnaire in a face-to-face interview. Data were collected using Kobo Toolbox, a reliable and user-friendly tool that can be utilized on smartphones [29].

### Statistical analysis

Palestinian women are first invited to undergo BC screening at the age of 40 [30]. Therefore, this cutoff was utilized to dichotomize the continuous variable of age into two categories: 18–39 years and ≥ 40 years. In addition, the minimum wage in Palestine is 1450 NIS (about $450) [31], therefore, this cutoff was utilized to dichotomize the continuous variable of monthly income into two categories: < 1450 NIS and ≥ 1450 NIS.

Descriptive statistics were used to summarize participant characteristics. Frequencies and percentages were used to describe categorical variables, while the median and interquartile range (IQR) were used to describe continuous variables with non-normal distribution. Kruskal–Wallis or Pearson’s Chi-square test was used to compare baseline characteristics of participants from the Gaza Strip versus those from the WBJ if they were continuous or categorical, respectively.

The assessed myths around BC causation were classified into food-related and food-unrelated myths. Frequencies and percentages were used to describe the recognition of each myth, and Pearson's Chi-square test was used to compare the recognition of participants from the Gaza Strip to that of those from the WBJ. Pearson’s Chi-square test was also used to compare the awareness level between participants from the Gaza Strip versus those from the WBJ.

Multivariable logistic regression analyses were utilized to examine the association between participant characteristics and recognizing each myth around BC causation. The multivariable analyses adjusted for age-group, educational level, occupation, monthly income, marital status, place of residency, having a chronic disease, knowing someone with cancer, and site of data collection. This model was determined a priori based on previous studies [8, 9, 12, 14, 28, 32–34].

Multivariable logistic regression analysis was also utilized to examine the association between participant characteristics and displaying good awareness of BC causation myths. The same aforementioned multivariable model was used.

Missing data were hypothesized to be missed completely at random and thus, complete case analysis was utilized to handle them. Data were analyzed using Stata software version 17.0 (StataCorp, College Station, Texas Gaza, United States).

## Results

### Characteristics of participants

Out of 6269 potential participants approached, 5434 completed the questionnaire (response rate: 86.7%), and 5257 were included in the final analysis (164 had missing data and 13 did not match the inclusion criteria): 2551 were from the Gaza Strip and 2706 were from the WBJ. Participants from the WBJ were older, earned higher monthly income, and suffered from more frequent chronic illnesses than those from the Gaza Strip (Table 1).

**Table 1:** Characteristics of study participants

Characteristic	Total (*n* = 5257)	Gaza Strip (*n* = 2551)	WBJ (*n* = 2706)	*p*-value
**Age**, median [IQR]	31.0 [24.0, 43.0]	30.0 [24.0, 40.0]	33.0 [24.0, 45.0]	< 0.001
**Age group**, n (%)				
18 to 39	3615 (68.8)	1859 (72.9)	1756 (64.9)	< 0.001
40 or older	1642 (31.2)	692 (27.1)	950 (35.1)	
**Educational level**, n (%)				
Secondary or below	3030 (57.6)	1457 (57.1)	1573 (58.1)	0.46
Post-secondary	2227 (42.4)	1094 (42.9)	1133 (41.9)	
**Occupation**, n (%)				
Unemployed/housewife	3581 (68.1)	1873 (73.4)	1708 (63.1)	< 0.001
Employed	1052 (20.0)	380 (14.9)	672 (24.9)	
Student	624 (11.9)	298 (11.7)	326 (12.0)	
**Monthly income ≥ 1450 NIS**, n (%)	3055 (58.1)	716 (28.1)	2339 (86.4)	< 0.001
**Having a chronic disease**, n (%)	1058 (20.1)	397 (15.6)	661 (24.4)	< 0.001
**Knowing someone with cancer**, n (%)	2520 (47.9)	1083 (42.5)	1437 (53.1)	< 0.001
**Marital status**, n (%)				
Single	1301 (24.7)	626 (24.5)	675 (24.9)	< 0.001
Married	3658 (69.6)	1812 (71.0)	1846 (68.2)	
Divorced/widowed	298 (5.7)	113 (4.5)	185 (6.9)	
**Site of data collection**, n (%)				
Public spaces	1821 (34.6)	809 (31.7)	1012 (37.4)	< 0.001
Hospitals	2116 (40.3)	919 (36.0)	1197 (44.2)	
Primary healthcare centers	1320 (25.1)	823 (32.3)	497 (18.4)	

*n* number of participants, *IQR* interquartile range, *WBJ* West Bank and Jerusalem

### Recognition of BC causation myths

Regarding myths related to food, there were fewer respondents who answered ‘disagree’ or ‘strongly disagree’ than food-unrelated myths. ‘Eating burnt food’ was the most recognized food-related myth (n = 1414, 26.9%) followed by ‘drinking from plastic bottles’ (*n* = 1317, 25.1%) (Table 2). ‘Eating food containing additives’ was the least recognized food-related myth (*n* = 599, 11.4%). ‘Having a physical trauma’ was the most recognized food-unrelated myth (n = 2795, 53.2%), whereas the least recognized was ‘wearing tight bra’ (*n* = 1018, 19.4%).

**Table 2:** Summary of the assessment of public beliefs in mythical causes of breast cancer

**Mythical cause**	**Total** **(*n* = 5257)** n (%)	**Gaza Strip** **(*n* = 2551)** n (%)	**WBJ** **(*n* = 2706)** n (%)	***p*–value**
**Food-related myths**				
Eating burnt food (e.g., bread or barbeque)	1414 (26.9)	525 (20.6)	889 (32.9)	< 0.001
Drinking from plastic bottles	1317 (25.1)	680 (26.7)	637 (23.5)	0.009
Eating food containing artificial sweeteners (e.g., saccharine)	1106 (21.0)	576 (22.6)	530 (19.6)	0.008
Using microwave ovens	837 (15.9)	388 (15.2)	449 (16.6)	0.17
Eating genetically modified food (e.g., hybrid vegetables)	707 (13.5)	355 (13.9)	352 (13.0)	0.33
Eating food containing additives	599 (11.4)	275 (10.8)	324 (12.0)	0.17
**Food-unrelated myths**				
Having a physical trauma	2795 (53.2)	1311 (51.4)	1484 (54.8)	0.012
Using aerosol containers	2070 (39.4)	961 (37.7)	1109 (41.0)	0.014
Using cleaning products	1747 (33.2)	911 (35.7)	836 (30.9)	< 0.001
Feeling stressed	1634 (31.1)	832 (32.6)	802 (29.6)	0.020
Exposure to electromagnetic frequencies (e.g., Wi-Fi and Radio/TV frequencies)	1450 (27.6)	663 (26.0)	787 (29.1)	0.012
Living near power lines	1238 (23.6)	618 (24.2)	620 (22.9)	0.26
Using deodorants	1222 (23.3)	571 (22.4)	651 (24.1)	0.15
Using mobile phones	1115 (21.2)	542 (21.2)	573 (21.2)	0.95
Wearing tight bra	1018 (19.4)	498 (19.5)	520 (19.2)	0.78

Answers with ‘disagree’ or ‘strongly disagree’ were considered correct; all other answers were considered incorrect

*n* number of participants, *WBJ* West Bank and Jerusalem

### Good awareness of BC causation myths and its associated factors

Only 269 participants (5.1%) demonstrated good awareness of BC causation myths (i.e., responding with ‘disagree’ or ‘strongly disagree’ to more than 10 out of 15 BC mythical causes) (Table 3). Participants from both the WBJ and the Gaza Strip had similar likelihood to display good awareness (5.1% vs. 5.1%). On the multivariable analysis, having a chronic disease as well as visiting hospitals and primary healthcare centers were all associated with a decrease in the likelihood of displaying good awareness of BC causation myths (Table 4).

**Table 3:** Awareness level of breast cancer mythical causes among study participants

**Level**	**Total** n (%)	**Gaza Strip** n (%)	**WBJ** n (%)	***p*–value**
**Poor** (0–5 myths)	3771 (71.7)	1837 (72.0)	1934 (71.5)	0.88
**Fair** (6–10 myths)	1217 (23.2)	583 (22.9)	634 (23.4)	
**Good** (11–15 myths)	269 (5.1)	131 (5.1)	138 (5.1)	

Answers with ‘disagree’ or ‘strongly disagree’ were considered correct; all other answers were considered incorrect

*n* number of participants, *WBJ* West Bank and Jerusalem

**Table 4:** Bivariable and multivariable logistic regression analyzing factors associated with having good recognition of the mythical causes of breast cancer

Characteristic	Good recognition					
	**No (*N* = 4988)** n (%)	**Yes (*N* = 269)** n (%)	**COR (95% CI)**	***p*-value**	**AOR (95% CI)**^a^	***p*-value**
**Age group**						
18 to 39	3410 (68.4)	205 (76.2)	Ref	Ref	Ref	Ref
40 or older	1578 (31.6)	64 (23.8)	0.67 (0.51- 0.90)	0.007	0.78 (0.56- 1.10)	0.16
**Educational level**						
Secondary or below	2874 (57.6)	156 (58.0)	Ref	Ref	Ref	Ref
Post-secondary	2114 (42.4)	113 (42.0)	0.98 (0.77- 1.26)	0.90	0.79 (0.59- 1.06)	0.11
**Occupation**						
Housewife	3411 (68.4)	170 (63.2)	Ref	Ref	Ref	Ref
Employed	998 (20.0)	54 (20.1)	1.09 (0.79- 1.48)	0.61	0.98 (0.68- 1.40)	0.90
Student	579 (11.6)	45 (16.7)	1.56 (1.11- 2.19)	0.01	1.11 (0.71- 1.73)	0.66
**Monthly income**						
< 1450 NIS	2081 (41.7)	121 (45.0)	Ref	Ref	Ref	Ref
≥ 1450 NIS	2907 (58.3)	148 (55.0)	0.88 (0.68- 1.12)	0.29	0.79 (0.56- 1.09)	0.15
**Marital status**						
Single	1215 (24.4)	86 (32.0)	Ref	Ref	Ref	Ref
Married	3492 (70.0)	166 (61.7)	0.67 (0.51- 0.88)	0.004	0.89 (0.63- 1.27)	0.53
Divorced/Widowed	281 (5.6)	17 (6.3)	0.85 (0.50- 1.46)	0.57	1.15 (0.64- 2.10)	0.64
**Residency**						
Gaza Strip	2420 (48.5)	131 (48.7)	Ref	Ref	Ref	Ref
WBJ	2568 (51.5)	138 (51.3)	0.99 (0.78- 1.27)	0.95	1.16 (0.84- 1.60)	0.38
**Having a chronic disease**						
No	3966 (79.5)	233 (86.6)	Ref	Ref	Ref	Ref
Yes	1022 (20.5)	36 (13.4)	0.60 (0.42- 0.86)	0.005	0.67 (0.45- 1.00)	0.050
**Knowing someone with cancer**						
No	2601 (52.1)	136 (50.6)	Ref	Ref	Ref	Ref
Yes	2387 (47.9)	133 (49.4)	1.07 (0.83- 1.36)	0.61	1.06 (0.82- 1.36)	0.65
**Site of data collection**						
Public Spaces	1687 (33.8)	134 (49.8)	Ref	Ref	Ref	Ref
Hospitals	2037 (40.8)	79 (29.4)	0.49 (0.37- 0.65)	< 0.001	0.52 (0.38- 0.70)	< 0.001
Primary healthcare centers	1264 (25.4)	56 (20.8)	0.56 (0.40- 0.77)	< 0.001	0.57 (0.41- 0.81)	0.001

Answers with ‘disagree’ or ‘strongly disagree’ were considered correct; all other answers were considered incorrect

*COR* crude odds ratio, *AOR* adjusted odds ratio, *CI* confidence interval, *WBJ* West Bank and Jerusalem

^a^Adjusted for age-group, educational level, occupation, monthly income, marital status, residency, having a chronic disease, knowing someone with cancer, and site of data collection

### Association between participant characteristics and recognizing food-related myths about BC causation

Participants recruited from hospitals or primary healthcare centers were less likely than those recruited from public spaces to recognize all BC food-related myths (Table 5). In addition, older participants (≥ 40 years) were less likely than younger participants to recognize all BC food-related myths except ‘eating food containing artificial sweeteners’ and ‘using microwave ovens,’ where no associated differences were found. Furthermore, participants who had chronic diseases were less likely than those who did not to recognize half of BC food-related myths. Education level was not associated with recognition of BC food-related myths except for ‘eating genetically modified food’ and ‘eating food containing additives’, where participants with higher education (i.e., post-secondary) were less likely to recognize them (OR = 0.61, 95% CI: 0.50–0.73 and OR = 0.67, 95% CI: 0.55–0.83, respectively).

**Table 5:** Multivariable logistic regression analyzing factors associated with the recognition of each food-related mythical cause of breast cancer

**Characteristic**	**Eating burnt food**				**Drinking from plastic bottles**			
	**No (*N* = 3843)** **n (%)**	**Yes (*N* = 1414)** **n (%)**	**AOR (95% CI)**^**a**^	***p*-value**	**No (*N* = 3940)** **n (%)**	**Yes (*N* = 1317)** **n (%)**	**AOR (95% CI)**^**a**^	***p*-value**
**Age group**								
18 to 39	2557 (66.5)	1058 (74.8)	Ref	Ref	2638 (67.0)	977 (74.2)	Ref	Ref
40 or older	1286 (33.5)	356 (25.2)	0.64 (0.55- 0.76)	< 0.001	1302 (33.0)	340 (25.8)	0.78 (0.66- 0.93)	0.005
**Educational level**								
Secondary or below	2258 (58.8)	772 (54.6)	Ref	Ref	2301 (58.4)	729 (55.4)	Ref	Ref
Post–secondary	1585 (41.2)	642 (45.4)	0.96 (0.83- 1.11)	0.55	1639 (41.6)	588 (44.6)	0.98 (0.85- 1.14)	0.81
**Occupation**								
Unemployed/housewife	2698 (70.2)	883 (62.4)	Ref	Ref	2725 (69.2)	856 (65.0)	Ref	Ref
Employed	740 (19.3)	312 (22.1)	1.01 (0.85- 1.21)	0.89	793 (20.1)	259 (19.7)	0.95 (0.79- 1.14)	0.55
Student	405 (10.5)	219 (15.5)	1.17 (0.92- 1.48)	0.21	422 (10.7)	202 (15.3)	1.07 (0.84- 1.35)	0.60
**Monthly income**								
< 1450 NIS	1724 (44.9)	478 (33.8)	Ref	Ref	1614 (41.0)	588 (44.6)	Ref	Ref
≥ 1450 NIS	2119 (55.1)	936 (66.2)	1.08 (0.92- 1.28)	0.35	2326 (59.0)	729 (55.4)	0.88 (0.74- 1.04)	0.13
**Marital status**								
Single	880 (22.9)	421 (29.8)	Ref	Ref	899 (22.8)	402 (30.5)	Ref	Ref
Married	2750 (71.6)	908 (64.2)	0.95 (0.79- 1.14)	0.56	2815 (71.4)	843 (64.0)	0.83 (0.69- 0.99)	0.041
Divorced/Widowed	213 (5.5)	85 (6.0)	1.18 (0.86- 1.61)	0.30	226 (5.8)	72 (5.5)	0.92 (0.66- 1.26)	0.59
**Residency**								
Gaza Strip	2026 (52.7)	525 (37.1)	Ref	Ref	1871 (47.5)	680 (51.6)	Ref	Ref
WBJ	1817 (47.3)	889 (62.9)	1.76 (1.49- 2.07)	< 0.001	2069 (52.5)	637 (48.4)	0.91 (0.78- 1.07)	0.27
**Having a chronic disease**								
No	3042 (79.2)	1157 (81.8)	Ref	Ref	3113 (79.0)	1086 (82.5)	Ref	Ref
Yes	801 (20.8)	257 (18.2)	0.99 (0.82- 1.18)	0.88	827 (21.0)	231 (17.5)	0.98 (0.82- 1.18)	0.85
**Knowing someone with cancer**								
No	2039 (53.1)	698 (49.4)	Ref	Ref	2026 (51.4)	711 (54.0)	Ref	Ref
Yes	1804 (46.9)	716 (50.6)	1.07 (0.94- 1.21)	0.30	1914 (48.6)	606 (46.0)	0.91 (0.80- 1.03)	0.14
**Site of data collection**								
Public Spaces	1209 (31.5)	612 (43.3)	Ref	Ref	1264 (32.1)	557 (42.3)	Ref	Ref
Hospitals	1569 (40.8)	547 (38.7)	0.76 (0.65- 0.88)	< 0.001	1647 (41.8)	469 (35.6)	0.70 (0.60- 0.81)	< 0.001
Primary healthcare centers	1065 (27.7)	255 (18.0)	0.55 (0.46- 0.66)	< 0.001	1029 (26.1)	291 (22.1)	0.65 (0.54- 0.77)	< 0.001
**Characteristic**	**Eating food containing artificial sweeteners**				**Using microwave ovens**			
	**No (*N* = 4151)** n (%)	**Yes (*N* = 1106)** n (%)	**AOR (95% CI)**^a^	***p*-value**	**No (*N* = 4420)** n (%)	**Yes (*N* = 837)** n (%)	**AOR (95% CI)**^a^	***p*-value**
**Age group**								
18 to 39	2823 (68.0)	792 (71.6)	Ref	Ref	2997 (67.8)	618 (73.8)	Ref	Ref
40 or older	1328 (32.0)	314 (28.4)	0.90 (0.76- 1.08)	0.26	1423 (32.2)	219 (26.2)	0.85 (0.70- 1.04)	0.11
**Educational level**								
Secondary or below	2380 (57.3)	650 (58.8)	Ref	Ref	2570 (58.1)	460 (55.0)	Ref	Ref
Post–secondary	1771 (42.7)	456 (41.2)	0.85 (0.72- 1.00)	0.06	1850 (41.9)	377 (45.0)	0.97 (0.81- 1.16)	0.73
**Occupation**								
Unemployed/housewife	2846 (68.6)	735 (66.5)	Ref	Ref	3058 (69.2)	523 (62.5)	Ref	Ref
Employed	852 (20.5)	200 (18.1)	0.89 (0.73- 1.09)	0.25	863 (19.5)	189 (22.6)	1.14 (0.92- 1.41)	0.24
Student	453 (10.9)	171 (15.4)	1.31 (1.01- 1.69)	0.041	499 (11.3)	125 (14.9)	1.00 (0.76- 1.33)	0.98
**Monthly income**								
< 1450 NIS	1702 (41.0)	500 (45.2)	Ref	Ref	1845 (41.7)	357 (42.7)	Ref	Ref
≥ 1450 NIS	2449 (59.0)	606 (54.8)	0.93 (0.78- 1.11)	0.44	2575 (58.3)	480 (57.3)	0.79 (0.65- 0.97)	0.024
**Marital status**								
Single	997 (24.0)	304 (27.5)	Ref	Ref	1037 (23.5)	264 (31.5)	Ref	Ref
Married	2928 (70.5)	730 (66.0)	1.09 (0.90- 1.34)	0.38	3130 (70.8)	528 (63.1)	0.83 (0.67- 1.03)	0.09
Divorced/Widowed	226 (5.5)	72 (6.5)	1.43 (1.02- 2.00)	0.036	253 (5.7)	45 (5.4)	0.84 (0.58- 1.23)	0.37
**Residency**								
Gaza Strip	1975 (47.6)	576 (52.1)	Ref	Ref	2163 (48.9)	388 (46.4)	Ref	Ref
WBJ	2176 (52.4)	530 (47.9)	0.83 (0.70- 0.99)	0.034	2257 (51.1)	449 (53.6)	1.24 (1.02- 1.50)	0.030
**Having a chronic disease**								
No	3280 (79.0)	919 (83.1)	Ref	Ref	3500 (79.2)	699 (83.5)	Ref	Ref
Yes	871 (21.0)	187 (16.9)	0.79 (0.65- 0.96)	0.020	920 (20.8)	138 (16.5)	0.85 (0.68- 1.07)	0.16
**Knowing someone with cancer**								
No	2154 (51.9)	583 (52.7)	Ref	Ref	2265 (51.2)	472 (56.4)	Ref	Ref
Yes	1997 (48.1)	523 (47.3)	0.95 (0.83- 1.09)	0.45	2155 (48.8)	365 (43.6)	0.77 (0.66- 0.90)	0.001
**Site of data collection**								
Public Spaces	1334 (32.1)	487 (44.0)	Ref	Ref	1445 (32.7)	376 (44.9)	Ref	Ref
Hospitals	1687 (40.6)	429 (38.8)	0.70 (0.60 -0.82)	< 0.001	1804 (40.8)	312 (37.3)	0.71 (0.59- 0.85)	< 0.001
Primary healthcare centers	1130 (27.3)	190 (17.2)	0.43 (0.35- 0.52)	< 0.001	1171 (26.5)	149 (17.8)	0.51 (0.41- 0.63)	< 0.001
**Characteristic**	**Eating genetically modified food**				**Eating food containing additives**			
	**No (*N* = 4550)** n (%)	**Yes (*N* = 707)** n (%)	**AOR (95% CI)**^a^	***p*-value**	**No (*N* = 4658)** n (%)	**Yes (*N* = 599)** n (%)	**AOR (95% CI)**^a^	***p*-value**
**Age group**								
18 to 39	3065 (67.4)	550 (77.8)	Ref	Ref	3143 (67.5)	472 (78.8)	Ref	Ref
40 or older	1485 (32.6)	157 (22.2)	0.58 (0.47- 0.73)	< 0.001	1515 (32.5)	127 (21.2)	0.59 (0.47- 0.76)	< 0.001
**Educational level**								
Secondary or below	2588 (56.9)	442 (62.5)	Ref	Ref	2664 (57.2)	366 (61.1)	Ref	Ref
Post–secondary	1962 (43.1)	265 (37.5)	0.61 (0.50- 0.73)	< 0.001	1994 (42.8)	233 (38.9)	0.67 (0.55- 0.83)	< 0.001
**Occupation**								
Unemployed/housewife	3121 (68.6)	460 (65.1)	Ref	Ref	3178 (68.2)	403 (67.3)	Ref	Ref
Employed	917 (20.2)	135 (19.1)	1.06 (0.83- 1.34)	0.66	950 (20.4)	102 (17.0)	0.73 (0.56- 0.95)	0.018
Student	512 (11.2)	112 (15.8)	1.19 (0.88- 1.60)	0.27	530 (11.4)	94 (15.7)	0.79 (0.57- 1.08)	0.13
**Monthly income**								
< 1450 NIS	1870 (41.1)	332 (47.0)	Ref	Ref	1949 (41.8)	253 (42.2)	Ref	Ref
≥ 1450 NIS	2680 (58.9)	375 (53.0)	0.77 (0.62- 0.95)	0.016	2709 (58.2)	346 (57.8)	0.93 (0.74- 1.17)	0.53
**Marital status**								
Single	1087 (23.9)	214 (30.3)	Ref	Ref	1094 (23.5)	207 (34.6)	Ref	Ref
Married	3206 (70.5)	452 (63.9)	0.98 (0.78- 1.23)	0.85	3299 (70.8)	359 (59.9)	0.60 (0.47- 0.76)	< 0.001
Divorced/Widowed	257 (5.6)	41 (5.8)	1.15 (0.77- 1.73)	0.49	265 (5.7)	33 (5.5)	0.81 (0.52- 1.25)	0.33
**Residency**								
Gaza Strip	2196 (48.3)	355 (50.2)	Ref	Ref	2276 (48.9)	275 (45.9)	Ref	Ref
WBJ	2354 (51.7)	352 (49.8)	1.05 (0.85- 1.29)	0.67	2382 (51.1)	324 (54.1)	1.22 (0.97- 1.52)	0.09
**Having a chronic disease**								
No	3596 (79.0)	603 (85.3)	Ref	Ref	3685 (79.1)	514 (85.8)	Ref	Ref
Yes	954 (21.0)	104 (14.7)	0.78 (0.61- 1.00)	0.049	973 (20.9)	85 (14.2)	0.75 (0.57- 0.98)	0.038
**Knowing someone with cancer**								
No	2363 (51.9)	374 (52.9)	Ref	Ref	2420 (52.0)	317 (52.9)	Ref	Ref
Yes	2187 (48.1)	333 (47.1)	0.95 (0.81- 1.12)	0.54	2238 (48.0)	282 (47.1)	0.98 (0.82- 1.17)	0.80
**Site of data collection**								
Public Spaces	1481 (32.5)	340 (48.1)	Ref	Ref	1556 (33.4)	265 (44.2)	Ref	Ref
Hospitals	1860 (40.9)	256 (36.2)	0.60 (0.50- 0.73)	< 0.001	1891 (40.6)	225 (37.6)	0.75 (0.61- 0.92)	0.005
Primary healthcare centers	1209 (26.6)	111 (15.7)	0.38 (0.30- 0.48)	< 0.001	1211 (26.0)	109 (18.2)	0.55 (0.43- 0.71)	< 0.001

Answers with ‘disagree’ or ‘strongly disagree’ were considered correct; all other answers were considered incorrect

*AOR* adjusted odds ratio, *CI* confidence interval, *WBJ* West Bank and Jerusalem

^a^Adjusted for age-group, educational level, occupation, monthly income, marital status, residency, having a chronic disease, knowing someone with cancer, and site of data collection

### Association between participant characteristics and recognizing food-unrelated myths about BC causation

Participants recruited from hospitals or primary healthcare centers were less likely than those recruited from public spaces to recognize eight out of nine food-unrelated myths as incorrect (Table 6). In contrast, employed participants were less likely than the unemployed/housewives to recognize five out of nine BC food-unrelated myths. In addition, participants with higher education were less likely to recognize ‘using cleaning products’ (OR = 0.85, 95% CI: 0.74–0.97), ‘exposure to electromagnetic frequencies’ (OR = 0.85, 95% CI: 0.73–0.98), and ‘wearing tight bra’ (OR = 0.68, 95% CI: 0.56–0.81) and had similar likelihoods to recognize five out of nine BC food-unrelated myths as compared to participants with lower education.

**Table 6:** Multivariable logistic regression analyzing factors associated with the recognition of each food-unrelated mythical cause of breast cancer

**Characteristic**	**Having a physical trauma**				**Using aerosol containers**			
	**No (*N* = 2462)** **n (%)**	**Yes (*N* = 2795)** **n (%)**	**AOR (95% CI)**^**a**^	***p*-value**	**No (*N* = 3187)** **n (%)**	**Yes (*N* = 2070)** **n (%)**	**AOR (95% CI)**^**a**^	***p*-value**
**Age group**								
18 to 39	1653 (67.1)	1962 (70.2)	Ref	Ref	2105 (66.0)	1510 (72.9)	Ref	Ref
40 or older	809 (32.9)	833 (29.8)	0.90 (0.78- 1.03)	0.14	1082 (34.0)	560 (27.1)	0.73 (0.63- 0.84)	< 0.001
**Educational level**								
Secondary or below	1480 (60.1)	1550 (55.5)	Ref	Ref	1861 (58.4)	1169 (56.5)	Ref	Ref
Post–secondary	982 (39.9)	1245 (44.5)	1.23 (1.08- 1.41)	0.002	1326 (41.6)	901 (43.5)	0.92 (0.80- 1.05)	0.20
**Occupation**								
Unemployed/housewife	1671 (67.9)	1910 (68.3)	Ref	Ref	2202 (69.1)	1379 (66.6)	Ref	Ref
Employed	496 (20.1)	556 (19.9)	0.84 (0.71- 0.98)	0.028	635 (19.9)	417 (20.1)	0.84 (0.71- 0.99)	0.038
Student	295 (12.0)	329 (11.8)	0.88 (0.71- 1.10)	0.26	350 (11.0)	274 (13.3)	0.77 (0.62- 0.96)	0.023
**Monthly income**								
< 1450 NIS	1087 (44.2)	1115 (39.9)	Ref	Ref	1398 (43.9)	804 (38.8)	Ref	Ref
≥ 1450 NIS	1375 (55.8)	1680 (60.1)	1.09 (0.95- 1.26)	0.22	1789 (56.1)	1266 (61.2)	1.23 (1.06- 1.43)	0.007
**Marital status**								
Single	627 (25.5)	674 (24.1)	Ref	Ref	709 (22.2)	592 (28.6)	Ref	Ref
Married	1685 (68.4)	1973 (70.6)	1.20 (1.02- 1.42)	0.025	2292 (71.9)	1366 (66.0)	0.81 (0.69- 0.96)	0.013
Divorced/Widowed	150 (6.1)	148 (5.3)	1.09 (0.83- 1.44)	0.54	186 (5.9)	112 (5.4)	0.88 (0.66- 1.18)	0.39
**Residency**								
Gaza Strip	1240 (50.4)	1311 (46.9)	Ref	Ref	1590 (49.9)	961 (46.4)	Ref	Ref
WBJ	1222 (49.6)	1484 (53.1)	1.12 (0.98- 1.29)	0.10	1597 (50.1)	1109 (53.6)	1.02 (0.88- 1.17)	0.83
**Having a chronic disease**								
No	1938 (78.7)	2261 (80.9)	Ref	Ref	2515 (78.9)	1684 (81.4)	Ref	Ref
Yes	524 (21.3)	534 (19.1)	0.92 (0.79- 1.08)	0.32	672 (21.1)	386 (18.6)	1.02 (0.86- 1.19)	0.86
**Knowing someone with cancer**								
No	1314 (53.4)	1423 (50.9)	Ref	Ref	1663 (52.2)	1074 (51.9)	Ref	Ref
Yes	1148 (46.6)	1372 (49.1)	1.08 (0.97- 1.21)	0.16	1524 (47.8)	996 (48.1)	0.96 (0.86- 1.08)	0.54
**Site of data collection**								
Public Spaces	813 (33.0)	1008 (36.1)	Ref	Ref	952 (29.9)	869 (42.0)	Ref	Ref
Hospitals	1028 (41.8)	1088 (38.9)	0.84 (0.73- 0.96)	0.009	1349 (42.3)	767 (37.0)	0.64 (0.55- 0.73)	< 0.001
Primary healthcare centers	621 (25.2)	699 (25.0)	0.89 (0.76- 1.03)	0.12	886 (27.8)	434 (21.0)	0.54 (0.46- 0.63)	< 0.001
**Characteristic**	**Using cleaning products**				**Feeling stressed**			
	**No (*N* = 3510)** n (%)	**Yes (*N* = 1747)** n (%)	**AOR (95% CI)**^a^	***p*-value**	**No (*N* = 3623)** n (%)	**Yes (*N* = 1634)** n (%)	**AOR (95% CI)**^a^	***p*-value**
**Age group**								
18 to 39	2324 (66.2)	1291 (73.9)	Ref	Ref	2479 (68.4)	1136 (69.5)	Ref	Ref
40 or older	1186 (33.8)	456 (26.1)	0.66 (0.57- 0.77)	< 0.001	1144 (31.6)	498 (30.5)	0.96 (0.82- 1.11)	0.57
**Educational level**								
Secondary or below	2013 (57.4)	1017 (58.2)	Ref	Ref	2058 (56.8)	972 (59.5)	Ref	Ref
Post–secondary	1497 (42.6)	730 (41.8)	0.85 (0.74- 0.97)	0.017	1565 (43.2)	662 (40.5)	0.88 (0.77- 1.01)	0.08
**Occupation**								
Unemployed/housewife	2377 (67.7)	1204 (68.9)	Ref	Ref	2445 (67.5)	1136 (69.5)	Ref	Ref
Employed	705 (20.1)	347 (19.9)	0.95 (0.80- 1.12)	0.51	757 (20.9)	295 (18.1)	0.83 (0.70- 0.99)	0.037
Student	428 (12.2)	196 (11.2)	0.70 (0.56- 0.88)	0.003	421 (11.6)	203 (12.4)	0.99 (0.79- 1.26)	0.96
**Monthly income**								
< 1450 NIS	1447 (41.2)	755 (43.2)	Ref	Ref	1493 (41.2)	709 (43.4)	Ref	Ref
≥ 1450 NIS	2063 (58.8)	992 (56.8)	1.11 (0.95- 1.30)	0.18	2130 (58.8)	925 (56.6)	1.06 (0.90- 1.23)	0.50
**Marital status**								
Single	847 (24.1)	454 (26.0)	Ref	Ref	902 (24.9)	399 (24.4)	Ref	Ref
Married	2451 (69.8)	1207 (69.1)	0.95 (0.80- 1.12)	0.53	2518 (69.5)	1140 (69.8)	1.15 (0.96- 1.37)	0.13
Divorced/Widowed	212 (6.1)	86 (4.9)	0.88 (0.65- 1.20)	0.43	203 (5.6)	95 (5.8)	1.22 (0.90- 1.65)	0.19
**Residency**								
Gaza Strip	1640 (46.7)	911 (52.1)	Ref	Ref	1719 (47.4)	832 (50.9)	Ref	Ref
WBJ	1870 (53.3)	836 (47.9)	0.76 (0.66- 0.88)	< 0.001	1904 (52.6)	802 (49.1)	0.83 (0.71- 0.96)	0.015
**Having a chronic disease**								
No	2771 (78.9)	1428 (81.7)	Ref	Ref	2872 (79.3)	1327 (81.2)	Ref	Ref
Yes	739 (21.1)	319 (18.3)	1.03 (0.87- 1.21)	0.76	751 (20.7)	307 (18.8)	0.88 (0.74- 1.04)	0.13
**Knowing someone with cancer**								
No	1831 (52.2)	906 (51.9)	Ref	Ref	1872 (51.7)	865 (52.9)	Ref	Ref
Yes	1679 (47.8)	841 (48.1)	1.03 (0.91- 1.15)	0.68	1751 (48.3)	769 (47.1)	0.93 (0.83- 1.05)	0.24
**Site of data collection**								
Public Spaces	1143 (32.6)	678 (38.8)	Ref	Ref	1173 (32.4)	648 (39.7)	Ref	Ref
Hospitals	1462 (41.7)	654 (37.4)	0.73 (0.64- 0.85)	< 0.001	1471 (40.6)	645 (39.5)	0.74 (0.64- 0.85)	< 0.001
Primary healthcare centers	905 (25.7)	415 (23.8)	0.70 (0.60- 0.82)	< 0.001	979 (27.0)	341 (20.8)	0.56 (0.47- 0.66)	< 0.001
**Characteristic**	**Exposure to electromagnetic frequencies**				**Living near power lines**			
	**No (*N* = 3807)** n (%)	**Yes (*N* = 1450)** n (%)	**AOR (95% CI)**^a^	***p*-value**	**No (*N* = 4019)** n (%)	**Yes (*N* = 1238)** n (%)	**AOR (95% CI)**^a^	***p*-value**
**Age group**								
18 to 39	2626 (69.0)	989 (68.2)	Ref	Ref	2720 (67.7)	895 (72.3)	Ref	Ref
40 or older	1181 (31.0)	461 (31.8)	0.95 (0.81- 1.11)	0.51	1299 (32.3)	343 (27.7)	0.86 (0.73- 1.02)	0.09
**Educational level**								
Secondary or below	2136 (56.1)	894 (61.7)	Ref	Ref	2336 (58.1)	694 (56.1)	Ref	Ref
Post–secondary	1671 (43.9)	556 (38.3)	0.85 (0.73- 0.98)	0.026	1683 (41.9)	544 (43.9)	1.02 (0.88–1.19)	0.76
**Occupation**								
Unemployed/housewife	2536 (66.6)	1045 (72.1)	Ref	Ref	2746 (68.3)	835 (67.4)	Ref	Ref
Employed	796 (20.9)	256 (17.7)	0.78 (0.65- 0.94)	0.009	816 (20.3)	236 (19.1)	0.88 (0.73- 1.06)	0.17
Student	475 (12.5)	149 (10.2)	0.74 (0.58- 0.95)	0.019	457 (11.4)	167 (13.5)	0.96 (0.75- 1.24)	0.77
**Monthly income**								
< 1450 NIS	1605 (42.2)	597 (41.2)	Ref	Ref	1677 (41.7)	525 (42.4)	Ref	Ref
≥ 1450 NIS	2202 (57.8)	853 (58.8)	1.02 (0.87- 1.20)	0.83	2342 (58.3)	713 (57.6)	1.00 (0.84- 1.18)	0.96
**Marital status**								
Single	963 (25.3)	338 (23.3)	Ref	Ref	962 (23.9)	339 (27.4)	Ref	Ref
Married	2621 (68.8)	1037 (71.5)	0.99 (0.82- 1.19)	0.90	2813 (70.0)	845 (68.3)	0.96 (0.80- 1.16)	0.69
Divorced/Widowed	223 (5.9)	75 (5.2)	0.84 (0.61- 1.15)	0.28	244 (6.1)	54 (4.4)	0.74 (0.52- 1.05)	0.09
**Residency**								
Gaza Strip	1888 (49.6)	663 (45.7)	Ref	Ref	1933 (48.1)	618 (49.9)	Ref	Ref
WBJ	1919 (50.4)	787 (54.3)	1.15 (0.98- 1.35)	0.08	2086 (51.9)	620 (50.1)	0.93 (0.79- 1.10)	0.41
**Having a chronic disease**								
No	3048 (80.1)	1151 (79.4)	Ref	Ref	3187 (79.3)	1012 (81.7)	Ref	Ref
Yes	759 (19.9)	299 (20.6)	0.97 (0.81- 1.15)	0.69	832 (20.7)	226 (18.3)	0.97 (0.80- 1.17)	0.74
**Knowing someone with cancer**								
No	1993 (52.4)	744 (51.3)	Ref	Ref	2084 (51.9)	653 (52.7)	Ref	Ref
Yes	1814 (47.6)	706 (48.7)	1.02 (0.90- 1.15)	0.78	1935 (48.1)	585 (47.3)	0.96 (0.84- 1.09)	0.54
**Site of data collection**								
Public Spaces	1307 (34.3)	514 (35.4)	Ref	Ref	1332 (33.1)	489 (39.5)	Ref	Ref
Hospitals	1495 (39.3)	621 (42.8)	0.95 (0.82- 1.10)	0.52	1637 (40.7)	479 (38.7)	0.81 (0.69- 0.94)	0.007
Primary healthcare centers	1005 (26.4)	315 (21.8)	0.74 (0.63- 0.88)	0.001	1050 (26.2)	270 (21.8)	0.68 (0.57- 0.81)	< 0.001
**Characteristic**	**Using deodorants**				**Using mobile phones**			
	**No (*N* = 4035)** n (%)	**Yes (*N* = 1222)** n (%)	AOR (95% CI)*	***p*-value**	**No (*N* = 4142)** n (%)	**Yes (*N* = 1115)** n (%)	AOR (95% CI)*	***p*-value**
**Age group**								
18 to 39	2758 (68.4)	857 (70.1)	Ref	Ref	2763 (66.7)	852 (76.4)	Ref	Ref
40 or older	1277 (31.6)	365 (29.9)	0.91 (0.77- 1.08)	0.27	1379 (33.3)	263 (23.6)	0.75 (0.62- 0.90)	0.002
**Educational level**								
Secondary or below	2312 (57.3)	718 (58.8)	Ref	Ref	2451 (59.2)	579 (51.9)	Ref	Ref
Post–secondary	1723 (42.7)	504 (41.2)	0.89 (0.76- 1.03)	0.13	1691 (40.8)	536 (48.1)	0.97 (0.83- 1.14)	0.74
**Occupation**								
Unemployed/housewife	2753 (68.2)	828 (67.8)	Ref	Ref	2918 (70.4)	663 (59.5)	Ref	Ref
Employed	820 (20.4)	232 (19.0)	0.87 (0.72- 1.05)	0.14	790 (19.1)	262 (23.5)	1.19 (0.98- 1.44)	0.07
Student	462 (11.4)	162 (13.2)	0.92 (0.71- 1.18)	0.50	434 (10.5)	190 (17.0)	1.12 (0.87- 1.43)	0.38
**Monthly income**								
< 1450 NIS	1701 (42.2)	501 (41.0)	Ref	Ref	1761 (42.5)	441 (39.6)	Ref	Ref
≥ 1450 NIS	2334 (57.8)	721 (59.0)	1.01 (0.85- 1.20)	0.88	2381 (57.5)	674 (60.4)	1.07 (0.89- 1.27)	0.48
**Marital status**								
Single	958 (23.7)	343 (28.1)	Ref	Ref	916 (22.1)	385 (34.5)	Ref	Ref
Married	2843 (70.5)	815 (66.7)	0.82 (0.68- 0.98)	0.034	2982 (72.0)	676 (60.6)	0.74 (0.61- 0.89)	0.001
Divorced/Widowed	234 (5.8)	64 (5.2)	0.76 (0.54- 1.05)	0.10	244 (5.9)	54 (4.9)	0.75 (0.53- 1.06)	0.11
**Residency**								
Gaza Strip	1980 (49.1)	571 (46.7)	Ref	Ref	2009 (48.5)	542 (48.6)	Ref	Ref
WBJ	2055 (50.9)	651 (53.3)	1.07 (0.91- 1.27)	0.41	2133 (51.5)	573 (51.4)	0.96 (0.81- 1.14)	0.67
**Having a chronic disease**								
No	3229 (80.0)	970 (79.4)	Ref	Ref	3265 (78.8)	934 (83.8)	Ref	Ref
Yes	806 (20.0)	252 (20.6)	1.09 (0.91- 1.31)	0.33	877 (21.2)	181 (16.2)	0.98 (0.80- 1.20)	0.87
**Knowing someone with cancer**								
No	2117 (52.5)	620 (50.7)	Ref	Ref	2135 (51.5)	602 (54.0)	Ref	Ref
Yes	1918 (47.5)	602 (49.3)	1.05 (0.92- 1.19)	0.49	2007 (48.5)	513 (46.0)	0.89 (0.77- 1.02)	0.09
**Site of data collection**								
Public Spaces	1335 (33.1)	486 (39.8)	Ref	Ref	1308 (31.6)	513 (46.0)	Ref	Ref
Hospitals	1647 (40.8)	469 (38.4)	0.79 (0.67- 0.92)	0.003	1774 (42.8)	342 (30.7)	0.58 (0.49- 0.68)	<0.001
Primary healthcare centers	1053 (26.1)	267 (21.8)	0.72 (0.60- 0.86)	<0.001	1060 (25.6)	260 (23.3)	0.70 (0.58- 0.84)	<0.001

**Characteristic**	**Wearing tight bra**			
	**No (*N* = 4239)** n (%)	**Yes (*N* = 1018)** n (%)	AOR (95% CI)*	***p*-value**
**Age group**				
18 to 39	2884 (68.0)	731 (71.8)	Ref	Ref
40 or older	1355 (32.0)	287 (28.2)	0.85 (0.71- 1.02)	0.08
**Educational level**				
Secondary or below	2384 (56.2)	646 (63.5)	Ref	Ref
Post–secondary	1855 (43.8)	372 (36.5)	0.68 (0.56- 0.81)	<0.001
**Occupation**				
Unemployed/housewife	2867 (67.6)	714 (70.1)	Ref	Ref
Employed	882 (20.8)	170 (16.7)	0.80 (0.65- 0.99)	0.042
Student	490 (11.6)	134 (13.2)	0.92 (0.71- 1.21)	0.57
**Monthly income**				
< 1450 NIS	1742 (41.1)	460 (45.2)	Ref	Ref
≥ 1450 NIS	2497 (58.9)	558 (54.8)	0.86 (0.71- 1.03)	0.10
**Marital status**				
Single	1024 (24.2)	277 (27.2)	Ref	Ref
Married	2972 (70.1)	686 (67.4)	0.88 (0.72- 1.08)	0.22
Divorced/Widowed	243 (5.7)	55 (5.4)	0.88 (0.62- 1.26)	0.49
**Residency**				
Gaza Strip	2053 (48.4)	498 (48.9)	Ref	Ref
WBJ	2186 (51.6)	520 (51.1)	1.09 (0.91- 1.30)	0.36
**Having a chronic disease**				
No	3356 (79.2)	843 (82.8)	Ref	Ref
Yes	883 (20.8)	175 (17.2)	0.79 (0.64- 0.97)	0.022
**Knowing someone with cancer**				
No	2196 (51.8)	541 (53.1)	Ref	Ref
Yes	2043 (48.2)	477 (46.9)	0.95 (0.82- 1.09)	0.48
**Site of data collection**				
Public Spaces	1403 (33.1)	418 (41.1)	Ref	Ref
Hospitals	1725 (40.7)	391 (38.4)	0.72 (0.61- 0.85)	<0.001
Primary healthcare centers	1111 (26.2)	209 (20.5)	0.59 (0.48- 0.72)	<0.001

Answers with ‘disagree’ or ‘strongly disagree’ were considered correct; all other answers were considered incorrect

*AOR* adjusted odds ratio, *CI* confidence interval, *WBJ* West Bank and Jerusalem

^a^Adjusted for age-group, educational level, occupation, monthly income, marital status, residency, having a chronic disease, knowing someone with cancer, and site of data collection

## Discussion

In this study, the overall awareness of BC causation myths was very low with only about 5% displaying good awareness (i.e., recognition of more than 10 out of 15 BC causation myths to be incorrect). There were no notable differences in awareness levels between the Gaza Strip and the WBJ. Participants with chronic diseases as well as visitors to hospitals or primary healthcare centers had a lower likelihood of displaying good awareness. Myths related to food were generally less recognized than food-unrelated myths. The most recognized food-related myths were ‘eating burnt food’ and ‘drinking from plastic bottles’, while the least recognized was ‘eating food containing additives. The most recognized food-unrelated myth was ‘having a physical trauma’, whereas the least recognized was ‘wearing tight bra’.

More than 60% of BC cases in Palestine are diagnosed at advanced stages despite the efforts of the Palestinian Ministry of Health and other organizations to improve early diagnosis and treatment [35]. Niksic and colleagues found out that low cancer awareness was associated with poor cancer survival [6]. Previous national studies from Palestine found that only 41.7% and 38.4% of Palestinian women displayed good BC symptom and risk factor awareness, respectively [8, 9]. The current study assessed the level of recognition of BC causation myths and misconceptions among Palestinian women since health beliefs may shape individuals’ health behavior [10]. Active endorsement of myths instead of established risk factors may cause confusion for the public and may negatively impact their health behavior [14]**,** hindering the efforts to mitigate cancer burden.

This study shows poor recognition of BC myths, which aligns with previous studies [10, 14, 28]. In the present study, 80.6% and 46.8% believed that wearing a tight bra and having a physical trauma could increase the risk for developing BC, respectively. Ryan and colleagues surveyed 748 Irish participants and found a lower proportion (29%) believing of wearing a tight bra as a BC risk factor but a similar proportion (48%) thinking so of a blow to the breast [10]. In addition, the authors found a high proportion of participants (86%) who believed that using mobile phones strongly increases the risk of cancer [10], similar to this study (78.8%). Moreover, Palestinian women could recognize the myths as incorrect causes of BC less than the actual risk factors of BC [8], similar to the results of a previous study that demonstrated the difficulty to distinguish the actual causes of cancer from mythical causes as a result of misinformation on the news and social networks [32].

Food related myths were less recognized as false causes of BC in this study. The relationship between diet and cancer is well-established. About one-third of cancer deaths could be attributed to diet and lifestyle [36], and 30 to 50% of all cancer cases are estimated to be preventable through healthy choices such as avoiding tobacco coupled with healthy diet and maintaining a normal body weight [37]. The Third Expert Report from the World Cancer Research Fund/American Institute for Cancer Research (WCRF/AICR) suggested that low intake of fruits and vegetables, high consumption of alcohol, and red processed meat are associated with increased cancer risk [37, 38]. National studies from Palestine found that 46.0% and 62.6% of participants recognized that being overweight and consuming fatty food could be risk factors for BC, respectively [8], and 42.9% and 53.4% recognized consuming red meat once or more per day and having a diet low in fibers as risk factors for colorectal cancer [22]. While in the present study, 88.6% and 86.5% of respondents believed that eating food containing additives and genetically modified food could increase BC risk. The discrepancy between the level of awareness of actual and mythical dietary factors can be problematic because dietary choices can affect health and the likelihood of developing BC [38].

At a time when the internet and social networks are considered a major source of health information, which is sometimes incorrect, the public now has access to information more easily than ever, and as a largely unregulated source for health information, myths about BC are still being replicated [39, 40]. According to the Palestinian Central Bureau of Statistics, 79.6% of the public in Palestine had internet access, 86.2% used social media, and 55.0% used the internet to seek health information in 2019 [41]. A previous study from Saudi Arabia found that 93.9% of the participants used at least one platform of social media to obtain health information, and 50.0% believed that the obtained health information from social media was reliable [42]. Johnson and colleagues reviewed 200 of the most popular social media articles about four of the most common cancers (breast, colorectal, lung, and prostate) posted on Facebook and other social networks, and they found that nearly one third of those articles contained misinformation, and 76.9% contained harmful information. The authors also found that engagement for articles with misinformation was greater than evidence-based articles [43]. Misinformation is thought to spread faster than true information because their content is usually novel and elicits more disgust, fear, or surprise [44]. The official website for the Palestinian Ministry of Health focuses on BC screening programs, early detection, signs and symptoms, and risk factors with no clear information addressing BC myths and misinformation [45]. Therefore, it is critical to make evidence-based information about BC causation available to Palestinian women with clear, evidence-based advice on how to reduce personal risks of BC. Such information may contribute to the efforts of women to make healthy lifestyle choices and prevent them from diverting their efforts to non-evidence-based interventions [46].

Surprisingly, in this study, visiting hospitals and primary healthcare centers was associated with lower likelihood of displaying good awareness as well as recognizing all BC causation myths. This contradicts with a previous study from Palestine that found that visiting governmental hospitals and primary healthcare centers was associated with an increase in likelihood of recognizing BC risk factors [8]. Higher education level and employment were also not associated with improved recognition of most BC myths. This was another unexpected finding, as postsecondary education was found to be associated with an increase in the likelihood of displaying good awareness of BC risk factors [8]. Health literacy is a distinct concept from literacy in general [47]. While the latter refers to being well-educated [47], health literacy specifically relates to an individual's ability to obtain, communicate, process, and understand basic health information and services necessary for making informed health decisions [48]. However, education is not the only factor that determines an individual's health literacy. Culture and society, the health system, and education are all crucial components of health literacy. These domains provide opportunities for intervention and improvement in health literacy, but they also present challenges [49]. Therefore, efforts to enhance health literacy, and hence health behavior, must consider the broader social and cultural contexts in which individuals live and access health information and services.

Additionally, we found that participants with chronic diseases were less likely to display good level of recognition of BC causation myths compared to those without such conditions. A previous national study from Palestine found that having a chronic disease was not associated with good awareness of BC risk factors [8]. Another previous study also demonstrated that participants with no chronic diseases held significantly more positive beliefs about cancer than those with poor/fair health [50]. These observations are especially important because many previous studies have shown that women with BC were more likely to die from cancer as well as all-cause mortality if they had other comorbidities [51–53], and that individuals who rated their health as fair or poor were more likely to have barriers to seeking healthcare [54]. This, combined with the negative association between low BC awareness and BC risk [55], can add to the cancer burden in this subgroup [53].

### Future directions

In the presence of low uptake of BC screening programs in Palestine [8], cancer prevention through public education on BC risk-reduction strategies and mitigating the stigma and myths surrounding the disease are essential for BC control. In fact, health promotion and early detection are the first pillar of the World Health Organization's Global Breast Cancer Initiative [56]. Despite numerous BC awareness campaigns in Palestine, there is a substantial need to promote Palestinian women’s knowledge about BC causation myths utilizing more innovative and culturally tailored methods. In addition, training healthcare professionals on how to educate women to be able to distinguish between evidence-based versus mythical BC causes is warranted [32]. Moreover, more information about BC mythical causes should be available to the Palestinian public through reliable websites such as the website of the Ministry of Health. Finally, university and school curricula should be enriched with more materials about well-established BC risk factors.

### Strengths and limitations

The main strengths of this study include the large sample size, the high response rate, and the wide coverage of the Palestinian community. This study has some limitations though. The exclusion of patients or visitors to oncology departments and those with medical backgrounds may have resulted in a smaller number of participants with higher awareness. Nevertheless, their exclusion was intended to ensure that the study accurately evaluated the public awareness of BC causation myths. A further limitation could be the use of convenience sampling that does not guarantee the generalizability of the study findings. Nonetheless, the high response rate coupled with the large sample size and the recruitment from various geographic locations may have mitigated this limitation. Moreover, our study did not capture data on some BC risk factors (e.g., smoking, high body mass index, older age at first childbirth..etc.) and assessing the awareness of BC causation myths in sub-groups based on these factors could be of interest. However, the primary scope of our study was to assess the overall public awareness of BC causation myths among Palestinian women. Finally, it was thought that participants recruited from hospitals and primary healthcare centers may have a health seeking behavior. In order to minimize the impact of this behavior on study findings, participants were also recruited from public spaces of the governorates corresponding to the included hospitals and primary healthcare centers. However, this may not be a perfect way to account for this. We also attempted to address this issue further by including the site of data collection (hospitals vs. primary healthcare centers vs. public spaces) in all our multivariable analyses. Notably, our findings indicate that participants recruited from hospitals and primary healthcare centers were less likely to demonstrate good awareness of BC causation myths.

## Conclusions

This study found a poor awareness of BC causation myths among Palestinian women with only 5% recognizing more than 10 out of 15 BC mythical causes. There were no notable differences in awareness levels between participants from the Gaza Strip and the WBJ. Having a chronic disease and visiting hospitals and primary healthcare centers were associated with a decrease in the likelihood of displaying good awareness. Myths related to food were generally less recognized than other food-unrelated myths. The results of this study suggest that there is a substantial need to include clear information about BC causation in future educational intervention beside focusing on BC screening, signs and symptoms, and risk factors.

### Supplementary Information

**Additional file 1:** **Supplementary Table 1.** Summary of the data collection sites included in the study.

## Data Availability

The dataset used and analyzed during the current study is available from the corresponding author on reasonable request.

## Abbreviations

BC: Breast cancer
LMICs: Low- and middle-income countries
WBJ: West Bank and Jerusalem
CAM-MYCS: Cancer awareness measure-mythical causes scale
CI: Confidence interval
OR: Odds ratio
