# Supplementary material for: Common myths and misconceptions about breast cancer causation among Palestinian women: a national cross-sectional study
Source: BMC Public Health. 2023 Nov 29;23:2370. doi: 10.1186/s12889-023-17074-9 (PMC10688078; doi:10.1186/s12889-023-17074-9)
Supplement: Supplementary file 1 — Additional file 1: Supplementary Table 1. Summary of the data collection sites included in the study. [file 12889_2023_17074_MOESM1_ESM.docx]

**Common Myths and Misconceptions About Breast Cancer Causation among Palestinian Women: A National Cross-sectional Study**

Mohamedraed Elshami, MD, MMSc^1,2^*, Islam Osama Ismail, MD^2,^*, Mohammed Alser, MD^3^, Ibrahim Al-Slaibi, MD^4^, Roba Jamal Ghithan^5^, Faten Darwish Usrof^6^, Malak Ayman Mousa Qawasmi^7^, Heba Mahmoud Okshiya^8^, Nouran Ramzi Shaban Shurrab^5^, Ibtisam Ismail Mahfouz^9^, Aseel AbdulQader Fannon^9^, Mona Radi Mohammad Hawa^10^, Narmeen Giacaman^5^, Manar Ahmaro^5^, Rula Khader Zaatreh^11^, Wafa Aqel AbuKhalil^5^, Noor Khairi Melhim^12^, Ruba Jamal Madbouh^5^, Hala Jamal Abu Hziema^9^, Raghad Abed-Allateef Lahlooh^5^, Sara Nawaf Ubaiat^5^, Nour Ali Jaffal^5^, Reem Khaled Alawna^5^, Salsabeel Naeem Abed^9^, Bessan Nimer Ali Abuzahra^5^, Aya Jawad Abu Kwaik^13^, Mays Hafez Dodin^5^, Raghad Othman Taha^5^, Dina Mohammed Alashqar^9^, Roaa Abd-al-Fattah Mobarak^5^, Tasneem Smerat, MSc^14^, Shurouq I. Albarqi, PharmB^15^, Nasser Abu-El-Noor, PhD^16#^, Bettina Bottcher, MD, PhD^9#^

*Contributed equally as a first co-author.

^#^Contributed equally as a senior co-author.

^1^Division of Surgical Oncology, Department of Surgery, University Hospitals Cleveland Medical Center, Cleveland, OH, USA.

^2^Ministry of Health, Gaza, Palestine.

^3^ United Nations Relief and Works Agency for Palestine Refugees (UNRWA), Gaza, Palestine.

^4^Almakassed Hospital, Jerusalem, Palestine.

^5^Faculty of Medicine, Al-Quds University, Jerusalem, Palestine.

^6^Department of a Medical Laboratory Sciences, Faculty of Health Sciences, Islamic University of Gaza, Gaza City, Palestine.

^7^Department of Medical Laboratory Sciences, Hebron University, Hebron, Palestine

^8^Al-Shifa Hospital, Gaza, Palestine.

^9^Faculty of Medicine, Islamic University of Gaza, Gaza, Palestine.

^10^Tulkarem Governmental Hospital, Tulkarem, Palestine.

^11^Caritas Baby Hospital, Bethlehem, Palestine.

^12^Department of Pharmacy, An-Najah National University, Nablus, Palestine.

^13^Faculty of Dentistry, Al-Quds University, Jerusalem, Palestine.

^14^Faculty of Medicine and Health Sciences, Palestine Polytechnic University, Hebron, Palestine.

^15^Faculty of Pharmacy, Al-Azhar University of Gaza, Gaza, Palestine.

^16^Faculty of Nursing, Islamic University of Gaza, Gaza, Palestine.

**Corresponding author**

Mohamedraed Elshami, MD, MMSc

Division of Surgical Oncology

University Hospitals Cleveland Medical Center

11100 Euclid Avenue, Cleveland, OH 44106
Phone: 832-245-6055

Email: [mohamedraed.elshami@gmail.com](mailto:mohamedraed.elshami@gmail.com)

| **Supplementary table 1:** Summary of the data collection sites included in the study. | | |
| --- | --- | --- |
| **Governorate** | **Hospital(s)** | **Primary healthcare center(s)** |
| Hebron | Alia Governmental Hospital | Hebron Directorate of Health |
| Nablus | Rafidia Governmental Hospital | Nablus Directorate of Health |
| Bethlehem | Beit Jala Governmental Hospital | Bethlehem Directorate of Health |
| Ramallah | Palestine Medical Complex | Ramallah Directorate of Health |
| Tulkarm | Tulkarm Governmental Hospital | Tulkarm Directorate of Health |
| Jenin | Jenin Governmental Hospital | Jenin Directorate of Health |
| Jerusalem | Makassed Charitable Hospital | East Jerusalem Directorate of Health |
| North of Gaza | Indonesian Hospital | Jabalia Primary Healthcare Center |
| Gaza | Alshifa Medical Complex | Al-Remal Primary Healthcare Center |
| Middle Zone | Alaqsa Martyrs Hospital | Deir Al-Balah Primary Healthcare Center |
| Khanyounis | Nasser Medical Complex  European Gaza Hospital | Khanyounis Primary Healthcare Center |
